# Prevention of intestinal obstruction reveals progressive neurodegeneration in mutant *TDP-43 (A315T)* mice

**DOI:** 10.1186/1750-1326-9-24

**Published:** 2014-06-17

**Authors:** Sarah Herdewyn, Carla Cirillo, Ludo Van Den Bosch, Wim Robberecht, Pieter Vanden Berghe, Philip Van Damme

**Affiliations:** 1 KU Leuven - University of Leuven, Department of Neurosciences, Experimental Neurology and Leuven Research Institute for Neuroscience and Disease (LIND), Leuven, Belgium; 2 VIB, Vesalius Research Center, Laboratory of Neurobiology, Leuven, Belgium; 3 University Hospitals Leuven, Department of Neurology, Leuven, Belgium; 4 Laboratory for Enteric NeuroScience (LENS), Translational Research for Gastrointestinal Disorders (TARGID), KU Leuven, Leuven, Belgium

**Keywords:** ALS, TDP43, Gastro-intestinal tract, Neurodegeneration, Enteric nervous system

## Abstract

**Background:**

Intraneuronal inclusions of TAR DNA-binding protein 43 (TDP-43) have been found in the majority of Amyotrophic Lateral Sclerosis (ALS) patients. Mutations in the gene encoding TDP-43 cause familial ALS. Transgenic mice expressing mutant TDP-43 with one such mutation (*TDP-43 (A315T)*) under control of the murine prion promoter develop motor symptoms, but their use is currently hampered by sudden death. We aimed to understand and overcome the cause of sudden death in *TDP-43 (A315T)* mice. Since intestinal obstruction was suspected to be the cause, intestinal motility of *TDP-43 (A315T)* mice was studied in an *ex-vivo* pellet propulsion assay. The effect on the enteric and motor phenotype was assessed, both in animals on normal chow or on a jellified fiber deprived diet, aimed at preventing intestinal obstruction.

**Results:**

The frequency of the propulsive motor complexes was significantly reduced in the colon of *TDP-43 (A315T)* compared to non transgenic (NTG) mice. Immunohistochemistry revealed significant enlargement in size and reduction in number of the nitric oxide synthase (NOS) neurons in the myenteric plexus of *TDP-43 (A315T)* mice. Prevention of intestinal obstruction by jellified food abolished sudden death, allowing the motor phenotype to develop and slowly progress with a more pronounced degeneration of upper and lower motor axons. A downregulation of endogenous TDP-43 mRNA and protein levels was observed prior to neurodegeneration.

**Conclusion:**

*TDP-43 (A315T)* mice suffer from intestinal dysmotility due to degeneration of NOS neurons in the myenteric plexus. Feeding the mice jellified food prevents sudden death and allows the motor phenotype to progress.

## Background

Transactive response DNA-binding protein (TDP-43) is involved in the pathogenesis of several neurodegerative disorders, primarily in Amyotrophic Lateral Sclerosis (ALS) and Frontotemporal Lobar Degeneration (FTLD) [1]. TDP-43 inclusions have also been observed in Alzheimer’s disease [2], Guam parkinsonism-dementia complex [3], Huntington’s disease [4] and Hippocampal sclerosis [2]. Physiologically, TDP-43 shuttles between the nucleus, where it regulates transcription and splicing, and the cytoplasm, where it has a role in RNA transport, mRNA stability and is a component of stress granules [1,5]. Most ALS patients show TDP-43 pathology on postmortem tissue: TDP-43 is abnormally ubiquitinated, phosphorylated, cleaved, translocated to the cytoplasm and found in aggregates in the (upper and lower) motor neurons [1]. About 2 to 5% of the familial ALS cases are due to mutations in the gene encoding TDP-43 with clustering of mutations in its C-terminal region [6-8]. To unravel the disease mechanisms of *TDP-43* induced neurodegeneration, several transgenic mouse models have been generated [9-18]. In 2009, Wegorzewska et al. [15] reported a mouse (available from The Jackson laboratory, stock number 010700) with overexpression of mutant TDP-43 (A315T) under control of the mouse prion promoter, leading to a threefold overexpression of TDP-43 in spinal cord and brain. These mice were reported to develop gait abnormalities from 3 to 4 months on, a swimming gait accompanied by weight loss at 4.5 months and to survive 154 ± 19 days. At the pathological level, there was a 20% loss of lower motor neurons and a 50% loss of upper motor axons in the spinal cord. However, we and others have observed a sudden death in these mice, prior to the development of full neurological symptoms [19-21].

The aim of this study was to identify the pathological mechanism behind this sudden death and provide a solution to allow the neurodegeration to develop further and render this transgenic mouse a useful model to study *TDP-43* induced neurodegeneration.

## Results

### Sudden death in *TDP-43 (A315T)* mice is due to intestinal dysfunction

**Figure 1:**
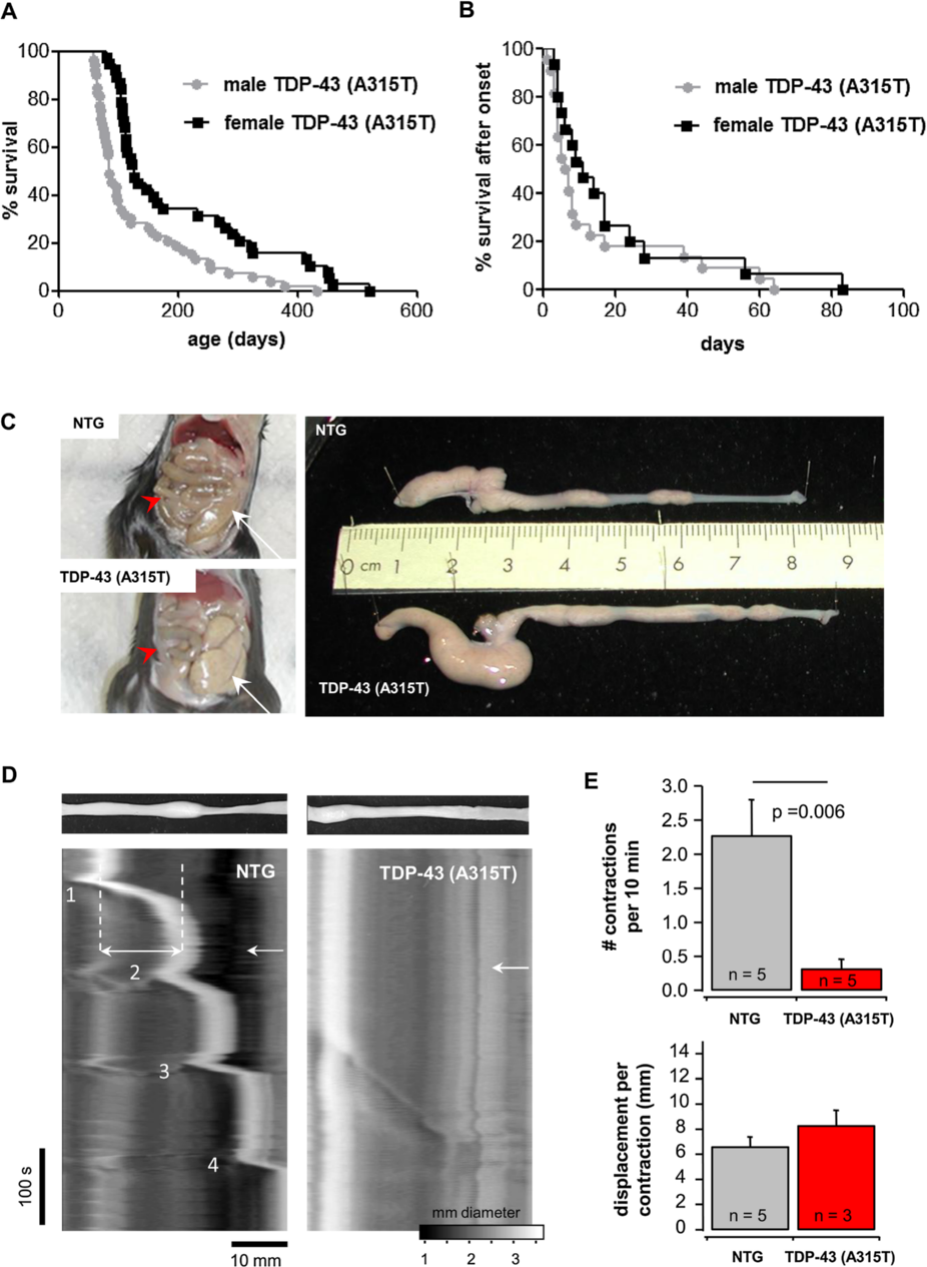
**Sudden death in *TDP-43 (A315T) *mice is due to intestinal dysfunction. A**: The survival of male *TDP-43 (A315T)* mice (median of 84 days, n = 53) differed significantly from female *TDP-43 (A315T)* mice (median of 126 days, n = 38) (p = 0.0004) and was for both genders very variable. **B**: Despite the variation in survival, the disease duration for both genders was very short: most mice died within a week after the onset of swimming gait abnormalities (median = 7 days for the males and median = 11 days for the females, n = 22 male and 15 female mice, p = 0.29). **C**: Comparison of intestines between NTG and *TDP-43 (A315T)* mice. *Left: TDP-43 (A315T)* mice displayed enlarged ileum (red arrowhead) and caecum (white arrow), which are easily detectable as compared to the normal-size ileum and colon from NTG mice. *Right:* Picture of the entire colon revealed no shortening in *TDP-43 (A315T)* compared to NTG mice. Note the absence of inflammatory patches or bleeding in the colon of *TDP-43 (A315T)* mice. **D**: Propulsion in colon segments from *TDP-43 (A315T)* and NTG mice. Recordings were made after the pellet was introduced in the colon (upper panels). Spatiotemporal map depicting the diameter of the colon segment in *TDP-43 (A315T)* (*right*) and NTG (*left*) mice (bar in x: 10 mm; bar in y: 100 s). **E**: The graphs show a significant reduction (p = 0.006) of frequency of contractions *(upper graph)* but not of force of muscle contraction *(lower graph)* between *TDP-43 (A315T)* (red bar) and NTG (grey bar) mice. *n* values indicate the number of mice from which the colon segments were analyzed.

We observed a sexual dimorphism in disease onset and survival of *TDP-43 (A315T)* mice, as reported previously [19]. Male mice had a median survival of 84 days, whereas females survived for 126 days (Figure 1A). This difference could not be explained by a difference in TDP-43 expression levels as assessed by Western blot analysis of spinal cord lysates. Although the disease onset was quite variable, most mice died within a week after onset of swimming gait abnormalities (Figure 1B). Contrary to mutant SOD1 mice, *TDP-43 (A315T)* mice died suddenly before the development of end stage (ES) motor symptoms. Sometimes, mice even died before the onset of neurologic abnormalities. These animals looked lethargic, a phenotype that is not directly compatible with ALS. Upon inspection, these mice appeared to have an extremely rigid abdomen indicative of intestinal obstruction, which was confirmed at autopsy. Even before the onset of motor symptoms, intestinal abnormalities with signs of pseudo-obstruction, thinned colon, enlarged caecum and distension of the small intestines could be observed (Figure 1C). The intestinal distension seemed most pronounced at the ileocaecal junction and was found to be progressive with age.

**Figure 2:**
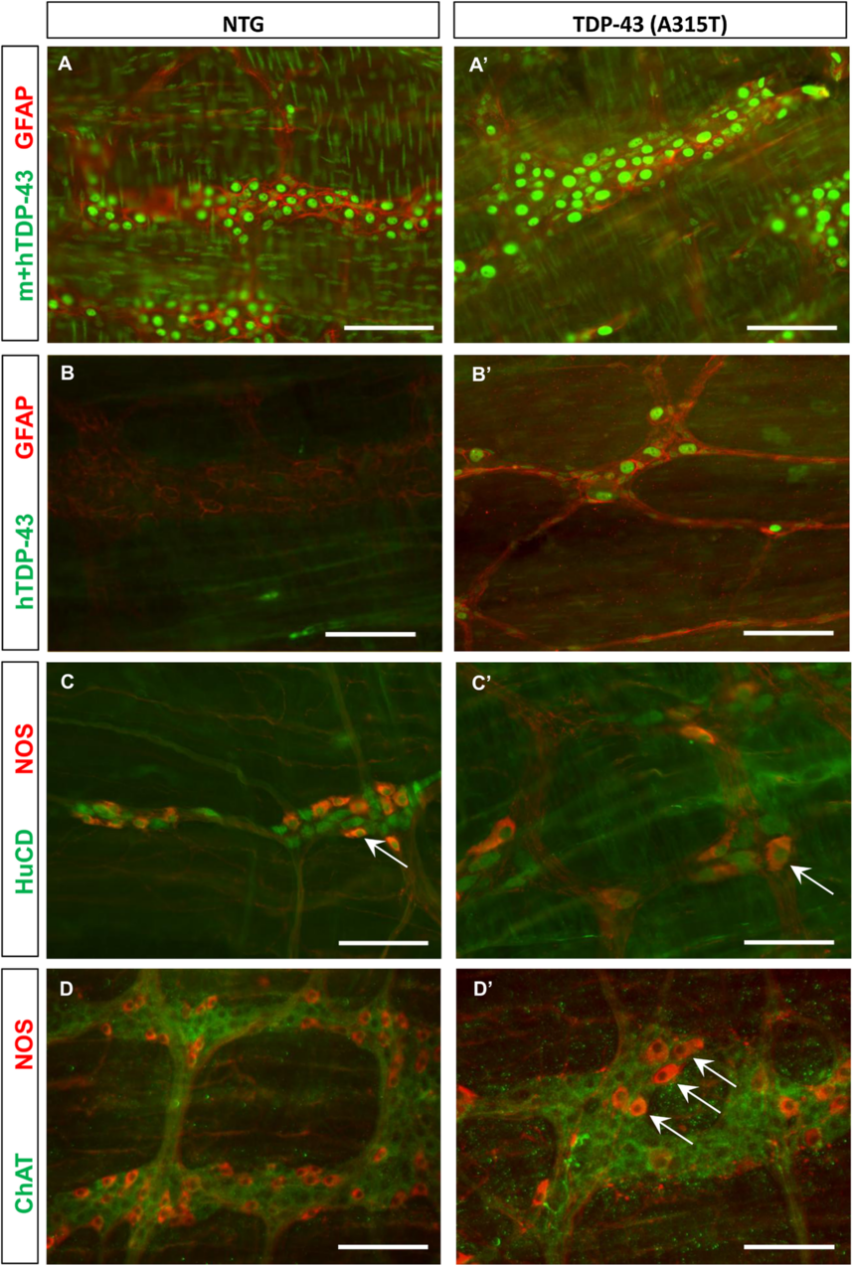
**Immunostaining of the myenteric plexus showed degeneration of NOS neurons in the Prnp *TDP-43 (A315T) *mice. A-A’**: Immunostaining of the myenteric plexus with TDP-43 antibody recognizing mouse and human TDP-43 (m + h, color code: green) revealed positive staining in the nuclei of all the cells both in NTG (A) and in *TDP-43 (A315T)* mice (A’). The GFAP staining already reveals a clear difference between the two genotypes: in A the healthy glia cell network clearly surrounds the enteric neurons, while in A’ the glial network is distorted. **B-B’**: Immunostaining of the myenteric plexus with a specific human TDP-43 antibody (hTDP-43, color code: green) confirmed specific expression of the transgene in the nuclei of neurons and glial cells (GFAP-positive, color code: red) in *TDP-43 (A315T)* mice (B’) while NTG mice were completely negative (B). **C-C’**: Immunostaining revealed significant reduction in number and enlargement in size of NOS neurons (white arrows, color code: red) in the myenteric plexus of *TDP-43 (A315T)* (C’) compared to NTG (C) mice. Total neurons were labeled with the panneuronal marker HuCD (C-C’; color code: green). **D-D’**: Immunostaining of the myenteric plexus with ChAT (cholinergic neurons, color code: green) and NOS (color code: red) revealed no significant differences in ChAT staining while again NOS neurons clearly appear fewer and larger (arrows) in *TDP-43 (A315T)* (D’) compared to NTG (D) mice. Scale bars: 100 μm.

To understand the mechanisms underlying the intestinal dysmotility, we performed video experiments to measure the intestinal propulsion (Figure 1D) and also examined the myenteric plexus by immunohistochemistry. The number of contractions induced by the presence of an artificial pellet inserted into the isolated colon was significantly reduced in *TDP-43 (A315T)* mice, compared to NTG littermates (Figure 1D and E, *upper graph*). The force of muscle contractions, as measured by the displacement of the pellet per contraction, was not affected in *TDP-43 (A315T)* mice compared to NTG (Figure 1D and E, *lower graph*), indicating that the peristaltic malfunction was of pure intrinsic neuronal origin. Immunostaining with a specific human TDP-43 antibody [22] confirmed the selective expression of the transgene in the nuclei of myenteric neurons (and glial cells), which is in line with a prion promoter (Prnp) pattern (Figure 2A and A’, 2B and B’). Although no major loss of enteric neurons was detected (HuCD positive neurons per ganglion: 37.1 ± 3.4 vs. 33.9 ± 2.5 for *TDP-43 (A315T)* and NTG mice, respectively), one specific population, the nitric-oxide synthase (NOS) expressing neurons, was severely affected in terminal ileum and colon. Their number was significantly reduced (8.4 ± 1.7 vs. 15.7 ± 1.9 per ganglion), and an enlarged swollen appearance of NOS neurons was noted (Figure 2C and C’), especially as mice got older. On the other hand, a staining for excitatory neurons, using a choline acetyltransferase (ChAT) antibody did not reveal any obvious differences between *TDP-43 (A315T)* and NTG mice (Figure 2D and D’). Furthermore, the enteric glial cell network that normally surrounds individual nerve cells was also severely distorted as shown by GFAP immunostaining (Figure 2A and A’, 2B and B’).

### Downregulation of endogenous TDP-43 at mRNA and protein level

**Figure 3:**
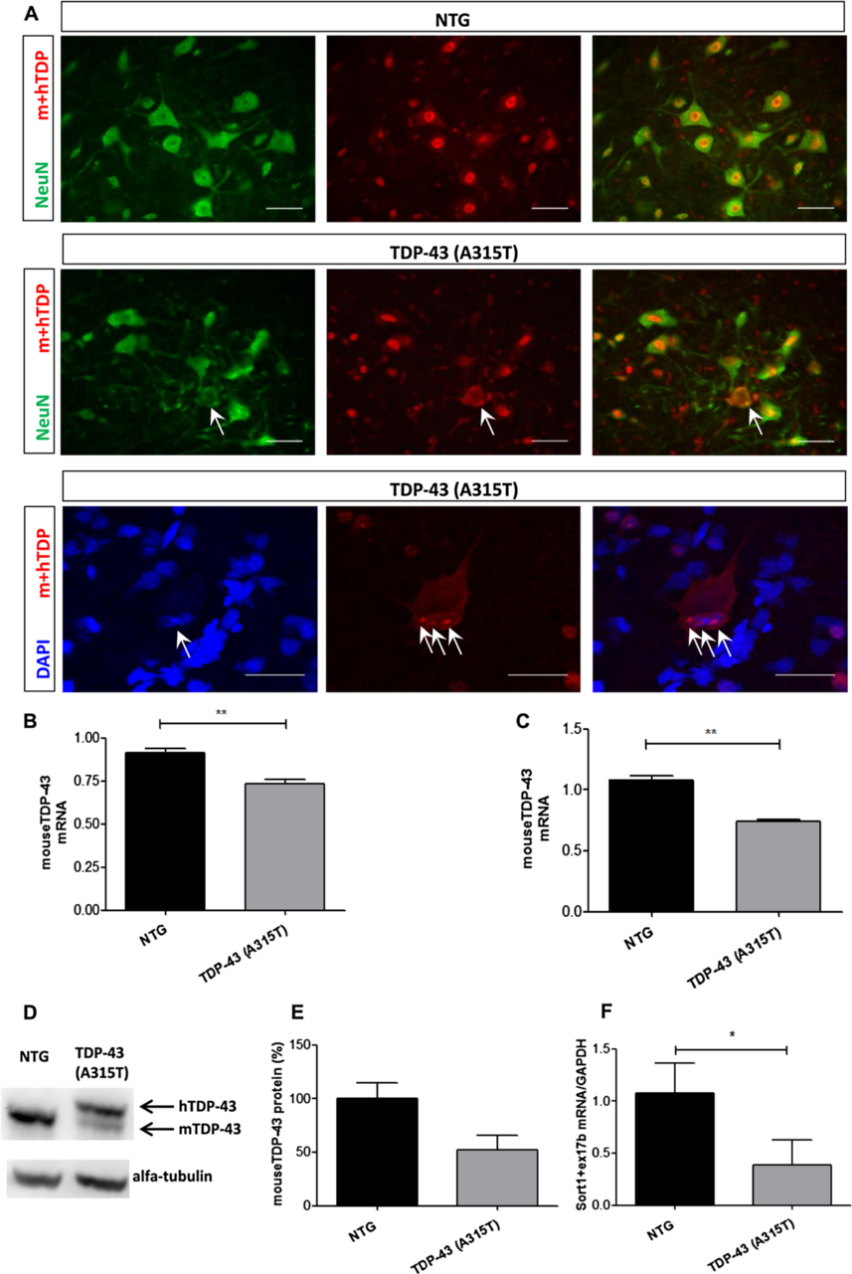
**Downregulation of endogenous TDP-43 at mRNA and protein level. A**: Immunostaining with the TDP-43 antibody that recognizes the C-terminal part of both mouse and human TDP-43 (12892-1-AP) of the ventral horn of the lumbar spinal cord of *TDP-43 (A315T)* mice at ES (*middle and lower panels*) compared to NTG mice (*upper panels*). Loss of nuclear TDP-43 (*middle panels*, arrows) and (intranuclear) TDP-43 inclusions (*lower panels*, arrows) could be detected, but were rare. For the pictures, both the separate images and the merged are shown (*red*: TDP-Ab; *green*: NeuN; *blue*: DAPI). Scale bars: 50 μm. **B**: 20% reduction of the endogenous mouse TDP-43 mRNA in the spinal cord due to the overexpression of exogenous human TDP-43 in the *TDP-43 (A315T)* mice (p < 0.0001, n = 3 for each genotype). **C**: 31% reduction of the endogenous mouse TDP-43 mRNA in the brain of *TDP-43 (A315T)* mice (p < 0.0001, n = 3 for each genotype). **D-E (D)** Representative Western blot of spinal cord tissue of NTG mice (*left*), compared to presymptomatic female *TDP-43 (A315T)* mice (*right*). The higher band, that is absent in the NTG mice, is the human TDP-43 protein, containing a Flag-tag. Alfa-tubulin was used for normalization (n = 3). **E**: Quantification showed a 48% loss of endogenous TDP-43. **F**: Downregulation of the levels of Sort1 with exon 17b inclusion in the brain of the *TDP-43 (A315T)* mice, compared to NTG mice (n = 3 for each genotype; p = 0.031).

Immunostainings using an antibody that recognizes the C-terminal part of both mouse and human TDP-43 (12892-1-AP) in NTG (Figure 3A, *upper panels*) and *TDP-43 (A315T)* mice (Figure 3A, *middle panels*), revealed that most neurons from *TDP-43 (A315T)* mice retain the nuclear TDP-43 expression, even at advanced disease stage. A (partial) loss of nuclear TDP-43 (Figure 3A, *middle panels*) and (intranuclear) TDP-43 inclusions (Figure 3A, *lower panels*) were only rarely seen in the *TDP-43 (A315T)* mice.

We could also detect a clear downregulation at the mRNA level of endogenous TDP-43 due to the overexpression of human mutant TDP-43 [10,23,24]. Using qPCR, we measured a 20% reduction of endogenous TDP-43 mRNA in the spinal cord of *TDP-43 (A315T)* mice compared to NTG littermates (Figure 3B) and a 31% reduction in the brain (Figure 3C). This downregulation of endogenous TDP-43 was also confirmed at the protein level by Western blot, where we detected a reduction of 48% in the spinal cord (Figure 3D and E).

To assess whether this downregulation of endogenous TDP-43 was associated with downstream splicing defects, we measured sortilin exon 17 b expression. In line with previous studies [25,26], we found a decrease in the levels of the splice variant of sortilin 1 with exon 17b inclusion (Sort1 + Ex17b) in the *TDP-43 (A315T)* mice (Figure 3F). Since it has been shown [25,26] that downregulation of TDP-43 in mouse N2a cells, leads to an increase in Sort1 + Ex17b levels, this suggests that the human TDP-43(A315T) overexpression can compensate for the downregulation of mouse TDP-43.

### Effect of gel food on disease progression and neurodegeneration in *TDP-43 (A315T)* mice

We sought to prevent the intestinal obstruction by removing non-digestible elements from the food and feeding the mice a nutrient gel (referred to as “gel” food). This diet was initiated before the development of gait abnormalities (at the age of 30 days for male and 80 days for female mice) and compared to the standard chow (referred to as “normal” food).

**Figure 4:**
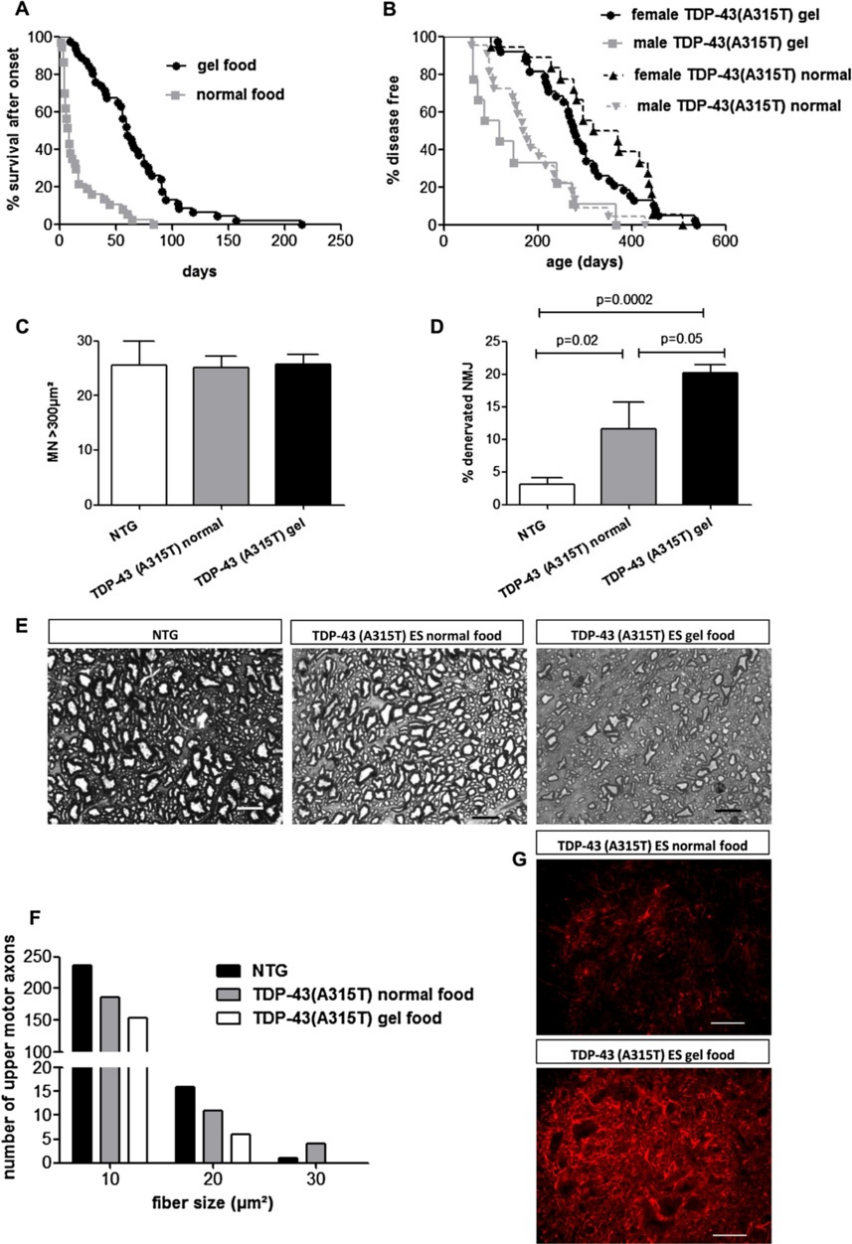
**Effect of gel food on neurodegeneration in *TDP-43(A315T) *mice. A**: The median disease duration increased for both genders from 8 days (d) with normal food (n=37) up to 60d (n=46) by gel food (p<0.0001). **B**: Disease onset is variable: 99-507d in the females (n=18, median=344d) with normal food, 58-427d in the males (n=22, median=172d); 114-538d (n=38, median=278d) in the females on gel food, 62-366d in the males (n=9, median=118d). The sexual dimorphism stays visible (overall p<0.0001), but there is no significant difference in onset between the mice on normal versus gel food for each gender. **C**: No difference (p=0.83) between the average number of motor neurons >300μm^2^ in the lumbar spinal cord for NTG (n=6) versus *TDP-43* mice with normal food (n=5) versus gel food (n=5) at ES. **D**: Significant increase in fully denervated NMJs at ES in the *TDP-43* mice on gel food, compared to *TDP-43* mice on normal food at ES and NTG mice, n=8/group. **E**: Gray scale images of the toluidine-blue staining of the white matter of the distal thoracic spinal cord, where the dorsolateral corticospinal tract axon fibers run, shown for a NTG mouse *(left)*, an ES *TDP-43* mouse with normal food *(middle)* and gel food *(right)*. Scale bars: 10μm. **F**: Histogram of results of the counting of the number of these corticospinal tract axons and their fiber size (n=4/group): the subsequent groups represent a fiber size of 5-15μm^2^ (bin center 10), 15-25μm^2^ (bin center 20) and 25–35μm^2^ (bin center 30). Statistical analysis showed a significant difference in number of fibers (p<0.0001 for comparison between all group with chi-square test and Bonferroni correction). **G**: Immunostaining with GFAP of the ventral horn of the spinal cord of a *TDP-43* mouse with normal food *(upper)* compared to gel food *(lower)* at ES. Scale bars: 50μm.

The effect of this simple intervention on the motor phenotype and survival of *TDP-43 (A315T)* mice was studied. First of all, gel feeding abolished the occurrence of sudden death. Instead, a slowly progressive motor phenotype was observed, resulting in an extended disease duration after the onset of obvious motor symptoms (Figure 4A). Both male and female mice developed more pronounced gait abnormalities with time (see Additional file 1: movies 1 and 2). After the onset of motor symptoms, we observed a slowly progressive weight loss and atrophy (of the gastrocnemius muscles). At ES, the mice were not able to turn from their side anymore and they had lost up to 40% of their body weight (see Additional file 1: movies 3 and 4). The disease onset remained extremely variable, ranging from 58 to 427 days in male and from 99 to 538 days in female mice (Figure 4B), but the survival after disease onset was extended with several weeks - even months - in the gel fed mice.

Histological analysis revealed a pathological substrate for the progressive motor phenotype. We could not find a lower motor neuron cell body loss when counting motor neurons in the ventral horn of the lumbar spinal cord (Figure 4C). The percentage of fully denervated neuromuscular junctions (NMJ) in the *TDP-43 (A315T)* mice was rather limited, but almost doubled in the longer surviving gel fed mice: 20% of denervated NMJ at ES in the *TDP-43 (A315T)* mice on gel food, 11.6% in the *TDP-43 (A315T)* mice with normal food at ES and 3% in NTG mice (Figure 4D). Also, gastrocnemius muscle atrophy was more pronounced. The serum CK levels at ES did not differ from NTG mice (p = 0.79). In addition, we could detect a more pronounced loss of upper motor axons in the lateral corticospinal tract of *TDP-43 (A315T)* mice receiving gel food [27] (Figure 4E). Especially, there was a more pronounced loss of larger axons (Figure 4F). We also observed an increased astrogliosis in the spinal cord of *TDP-43 (A315T)* mice put on gel food, compared to mice with normal food at ES (Figure 4G), illustrating the more advanced disease state reached.

**Figure 5:**
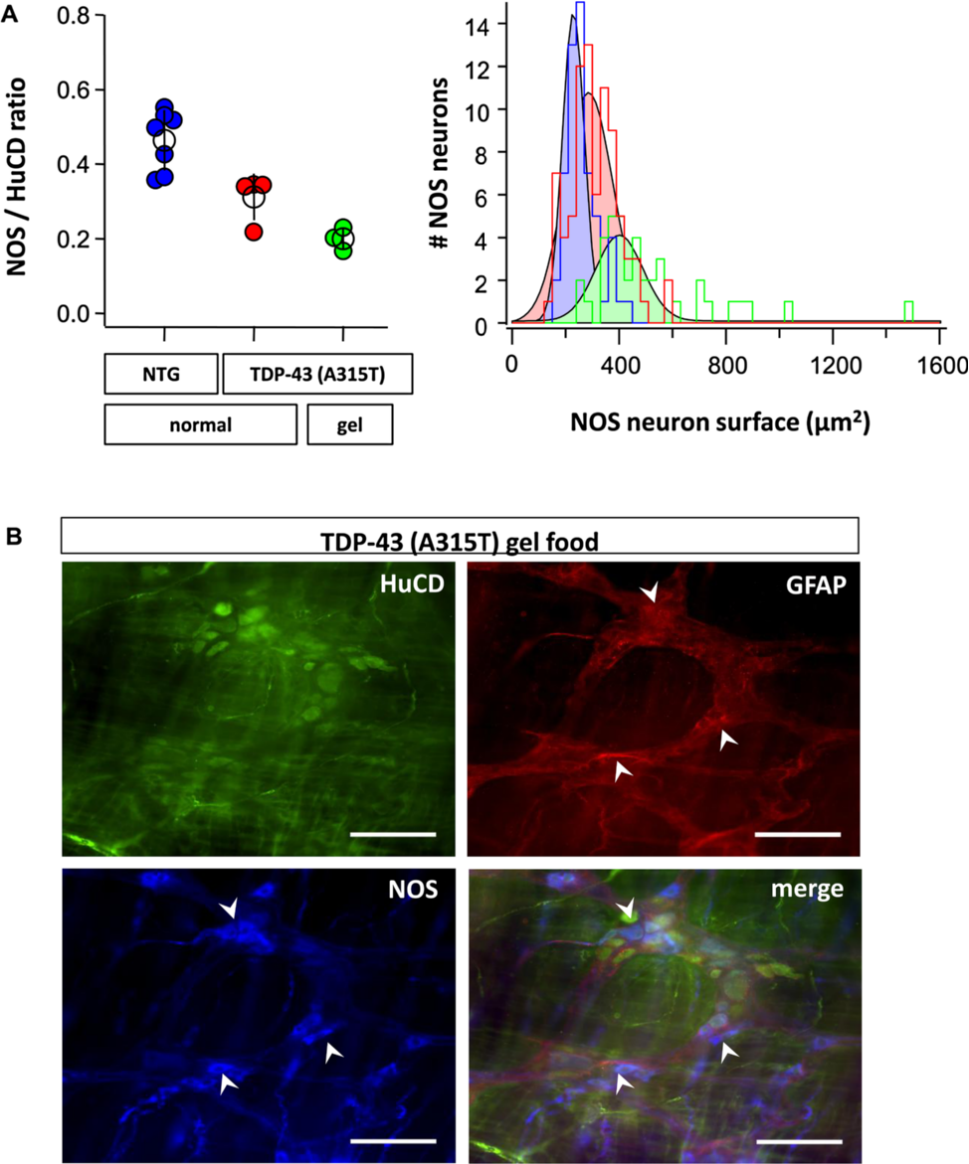
**Progressive loss of NOS neurons in *TDP-43 (A315T) *mice with gel food. A**: (*left*) The graph shows the NOS/HuCD neurons ratio in NTG (color code: blue; n = 7), *TDP-43 (A315T)* on normal food (color code: red; n = 4) and *TDP-43 (A315T)* on gel food (color code: green; n = 3) mice. (*right*) The histogram (and color matched Gaussian fits) displays the size of NOS neurons (in μm^2^) in NTG (color code: blue; n = 7), *TDP-43 (A315T)* normal fed (color code: red; n = 4) and *TDP-43 (A315T)* gel fed (color code: green; n = 3) mice, showing an enlargement of the remaining NOS neurons in *TDP-43 (A315T)* mice on normal food and even worse so in gel fed *TDP-43 (A315T)* mice. **B**: Immunostaining revealed enlargement of NOS neurons (white arrows; color code: blue) and distortion (white arrowheads) of glial network (GFAP in red) in *TDP-43 (A315T)* mice on gel food. Neurons were stained with panneuronal marker HuCD (color code: green). Scale bars: 100 μm.

To test whether the severe enteric nervous system (ENS) degeneration was progressing further in gel fed *TDP-43 (A315T)* mice, we performed immunostainings of the ENS. As can be appreciated in Figure 5A, the loss of enteric NOS neurons was also progressive in these animals, as numbers of NOS neurons were further reduced and remnant NOS neurons further enlarged. The enteric glial network was also more severely disrupted (Figure 5B). Taken together, feeding the *TDP-43 (A315T)* mice gel food abolished the sudden death and allowed a progressive motor phenotype to develop further.

## Discussion

To unravel the disease mechanisms of *TDP-43* induced neurodegeneration, several transgenic mouse models have been generated. Unfortunately, so far none of them fulfills the expectations. The *TDP-43 (A315T)* mice (designed by Wegorzewska et al. [15]) develop motor symptoms, but the sudden death, due to intestinal distension as reported previously [19-21] limits their use.

In this study we unraveled the nature of the intestinal problems in an *ex-vivo* pellet study, which revealed a significant reduction in the ability to generate propulsive contractions in these mice. Using immunohistochemical analysis, we observed a degeneration of NOS neurons in the ENS, which is responsible for the coordination of peristaltic movements. NOS neurons are responsible for inhibitory signaling within the ENS and together with excitatory neurons finely regulate intestinal peristalsis. Both excitation and inhibition are quintessential for luminal contents to be propelled. Lack of excitation would obviously fail to produce any contractile force, but also lack of inhibition prevents peristalsis as gut muscle relaxation in front of the luminal contents is equally important. In previous studies, the Prnp promoter was suggested to drive expression in particular in enteric glial cells [28]. However, we clearly observed a high transgene expression (of human mutant TDP-43) in the nuclei of neurons of the myenteric plexus. While writing this manuscript, an independent study [29] also described the transgene expression and neurodegeneration of the enteric neurons in this mouse model as an explanation for the reduced intestinal motility. The authors showed that mutant mice have a loss in acetylcholinesterase-positive neurons, which are excitatory, explaining, in part, the reduction in colonic propulsion. Here, we did not observe any difference in cholinergic neurons in *TDP-43 (A315T)* mice, but we mainly found a loss of NOS neurons, preceded by an increase in their cell soma size. These findings do not exclude each other. In fact, while the loss of NOS neurons may represent an early event, a later stage of the disease may be characterized by the impairment of cholinergic signaling as well, thus also affecting excitatory pathways. The degeneration of NOS neurons causes the loss of inhibitory control leading to abnormalities in intestinal propulsion, dysmotility and, finally, to pseudo-obstruction and sudden death. The exact mechanism that leads to the enlargement and loss of NOS neurons is not clear, but defects in the neuroprotective glial network may be involved, as demonstrated by GFAP distorted signaling. Also, reduced synaptic input could lead to loss of volume constraints in neurons. Medina et al. [21] reported a reduction in synaptophysin in the hippocampus of these mice, indicating a role of TDP-43 in synaptic function. Our study indicates that the ENS is vulnerable to the toxic effects of mutant TDP-43. This is in line with recent evidence showing parallel manifestations of various neuropathologies in the enteric and central nervous systems [30]. However, it remains largely unexplored if the ENS is affected in patients with ALS.

We could confirm the downregulation of endogenous TDP-43 in spinal cord and brain, both at mRNA and protein level, supporting a role for autoregulation of TDP-43 expression. TDP-43 can bind the 3’ UTR of its own transcript. This leads to decreased expression of endogenous TDP-43 due to the 3-fold overexpression of exogenous TDP-43 [15]. Since we and others [25,26] measured a decrease of Sort1 + Ex17b in the brain of human *TDP-43 (A315T)* overexpressing mice, in contrast to the upregulation seen in mouse N2a cells with TDP-43 knock-down, this suggests that the reduction of endogenous TDP-43 is overcompensated by the expression of human mutant *TDP-43 (A315T)*. As suggested previously [10,23,24], the downregulation of endogenous TDP-43 can contribute to the pathogenesis of neurodegeneration, but so does overcompensation, since a physiological range of expression, below or above which pathology develops, appears to be important. In line with previous studies [15,19], we could detect some loss of nuclear TDP-43 and (nuclear) TDP-43 inclusions in *TDP-43 (A315T)* mice at ES, but both were rare phenomena, suggesting that cytoplasmic mislocalization and inclusions are not the major cause of toxicity in this model.

Interestingly, a similar phenomenon of pseudo-obstruction was described in transgenic mice expressing CAG-repeats under the control of the Prnp [31]. Severely enlarged and loss of NOS neurons, together with intestinal pseudo-obstruction was seen in mice overexpressing the polyglutamine expansion responsible for SCA7. This suggests that the ENS is vulnerable to neurodegeneration as well, and may be a readily accessible part of the nervous system to study mechanisms of neurodegeneration, even in intestinal biopsies obtained from patients [32]. In addition, *TDP-43 (A315T)* mice could become a useful model to study intestinal motility disorders caused by degeneration, in addition to the developmental models of intestinal dysfunction.

In this study we succeeded to overcome the sudden death by feeding the mice gel food and thereby preventing intestinal constipation. This intervention significantly extended survival and allowed a progressive motor phenotype to develop. Disease progression was associated with a more pronounced loss of upper and lower motor axons. An important limitation of this model remains the lack of lower motor neuron degeneration in the ventral horn of the spinal cord. Compared to mutant *SOD1* mice, *TDP-43 (A315T)* mice have limited loss of NMJs. A dysfunctional neuromuscular transmission is not excluded in the current study and may contribute to the muscle weakness observed. However, the progressive motor phenotype observed can most likely be attributed to loss of upper motor neuron axons. Therefore, *TDP-43 (A315T)* mice can be of value in studying the upper motor neuron degeneration, that is an essential hallmark of ALS (with and without *TDP-43* mutations), but is less well studied so far. The large variation in disease onset makes this model less suited for cross breeding experiments, although a similar heterogeneity in disease onset is also apparent in ALS patients.

## Conclusions

In summary, feeding the *TDP-43 (A315T)* mice jellified fiber deprived food, abolished sudden death due to intestinal dysmotility and allowed the slowly progressive upper and lower motor axon phenotype to develop further, thus rendering this mouse a suitable model for *TDP-43* induced (upper motor) neurodegeneration.

## Materials and methods

### Feeding and follow-up of *TDP-43 (A315T)* mice

The *TDP-43 (A315T)* mice designed by Wegorzewska et al. [15] contain the mutant human *TDP-43* gene, preceded by the Flag-tag, under control of the mouse Prnp, leading to the highest expression in spinal cord and brain, but also in other tissues. Originally, these mice were on a mixed C57BL/6 J and CBA background. We obtained these mice from the Jackson Laboratory, stock number 010700 (The Jackson Laboratory, Bar Harbor, Maine, USA). The mice were already backcrossed for 10 generations into a C57BL/6 J background, leading to a pure C57BL/6 J background. For genotyping, we used the primers and protocol as previously described [20]. Mice were kept in a conventional facility and fed ad libitum with dry pellet food (Ssniff® R/M-H, Ssniff Spezialdiäten GmbH, Soest, Germany) for the non-transgenic (NTG) mice and the *TDP-43 (A315T)* mice with normal food. The gel fed mice got DietGel®boost (ClearH20, Maine, USA) from 30 days on for male and 80 days on for female mice. This gel food contains all necessary nutrients, but is a soft, high calorie, easily digestible paste with hardly any fibers.

The mice on gel food were put in cages, without bedding to prevent that wood fibers would stick to their food. These cages have a grid on the bottom, which allows the stool and urine to pass, but does not cause difficulties walking. Because of the high grade of humidity of the gel food, the fur of the mice looked wet. In some cages mice with the earliest disease onset, lost (part) of their fur (and whiskers) because of grazing by the others. This phenomenon does not affect their well-being and was reversible within 2 weeks after they were put alone in a cage.

NTG C57BL/6 J mice of both genders were compared to *TDP-43 (A315T)* mice with normal food and gel food for motor performance, body appearance and survival once a week till onset, than twice a week. Onset of gait abnormalities was defined as the moment at which a clear swimming gait appeared, with the paws wide-based and the animal being unable to hold its lower body from the ground. End stage (ES) for the *TDP-43 (A315T)* mice with normal food was the moment at which the animal appeared immobile (even after being gently pushed) and lethargic, since we experienced that in this stage the death would occur within 24 h. For the gel fed mice, ES was the moment at which the mouse could not turn from a side anymore within 30 s. At this point the mice were euthanized using a lethal dose of 10% Nembutal and tissues were collected.

### Ex-vivo pellet propulsion assay and video analysis of colonic function

*TDP-43 (A315T)* female mice of around 90 days were compared to NTG littermates of the same age (n = 5). After killing the mice by cervical dislocation, the entire proximal and distal colon were removed and suspended in an organ bath (±30 min after death) filled with Krebs solution (in mM: 120.9 NaCl, 5.9 KCl, 1.2 MgCl_2_, 2.5 CaCl_2_, 1.2 NaH_2_PO_4_, 14.4 NaHCO_3_, 11.5 glucose) kept at 37°C and continuously bubbled with 95% O_2_–5% CO_2_ (pH 7.4). The intestine was allowed to equilibrate for 30 min, after which an artificial pellet was gently introduced into the proximal colon. Due to the intrinsic peristaltic reflex, the pellet was pushed along the intestine in a consecutive series of individual contraction, which can be monitored using a video setup. All images were recorded using custom written routines in Igor pro (Wavemetrics, Eugene, OR) [33]. The position of the pellet can be monitored by the dilated diameter (top images) that is shown as a gray scale line in the spatio-temporal maps (white arrows) [34]. Frequency and propulsive force of the contraction can be deduced from the generated maps.

### Histopathologic analyses

Scoring of the NMJ was done as previously described [35]. Eight mice were analyzed in each group at 100–150 NMJs/mouse.

For the measurement of creatine kinase (CK) levels, blood was taken from the right atrium after euthanizing, but before perfusion of the mice (at ES for the *TDP-43 (A315T)* mice with normal or gel food). The sample was centrifuged for 10 minutes at 14000 rpm. The supernatant was pipetted into another eppendorf tube, which was again centrifuged for 10 minutes at 14000 rpm. This supernatant was stored at −80°C until all the samples were collected. CK levels were determined by the same technique as used for human samples at the laboratory of UZ Leuven, Belgium.

We performed immunostainings for total (mouse and human) TDP-43 and glial fibrillary acid protein (GFAP) on spinal cord of ES (normal or gel fed) *TDP-43 (A315T)* mice compared to NTG mice. To this end, mice were euthanized with 10% Nembutal and perfused with PBS and 4% PFA. Lumbar spinal cord was dissected and dehydrated overnight in 30% sucrose in PBS. The tissue was embedded in Tissue Tek (OCT Compound, 361603E, VWR International, Randor, Pennsylvania, USA) and frozen at −80°C. Slices of 20 μm were obtained by using a microtome (Slee cryostat, Mainz, Germany). For immunostainings, they were blocked with 10% normal donkey serum (Sigma Aldrich, St Louis, Missouri, USA) at room temperature, incubated overnight at 4°C with the C-terminal total (mouse and human) TDP-43 (polyclonal rabbit-anti-TDP43-Ab 12892-1-AP, Proteintech, 1/200), followed by incubation with NeuN-Ab (MAB377, Millipore, Billerica, Massachusetts, USA, 1/200 for 1 h at room temperature) or incubated for 2 hours at room temperature with GFAP-Ab (polyclonal rabbit anti-GFAP-Ab, DAKO, Glostrup, Denmark, 1/500) followed by 3 washes with PBS-T and incubation with the secondary Ab for 1 h at room temperature [1/500, Alexa Fluor 555 anti-rabbit (for TDP-43-Ab and GFAP-Ab) or Alexa Fluor 488 anti-mouse (for NeuN), Invitrogen Life Technologies, Carlsbad, CA, USA]. After 3 washes with PBS-T, slides were mounted with Vectashield with DAPI and analyzed using a Zeiss Imager M1 microscope (Zeiss, Oberkochen, Germany).

To analyze the intestinal tract by immunostainings, segments of intestine (ileum and colon) were collected, opened along the mesenteric border and pinned flat in a sylgard lined dissection dish. Using fine forceps the mucosal and submucosal layers were removed prior to fixation in 4% paraformaldehyde (30 min). After rinsing in PBS, circular muscle layers were peeled in case of the small intestine; while for the colon the longitudinal muscle was removed. Tissues were treated in permeabilizing (0.5% triton-x) and 4% goat/donkey serum, prior to a 24 h (4°C) incubation in primary antibodies: specific human TDP-43 antibody (monoclonal mouse anti-hTDP-43-Ab 60019-2-Ig, Proteintech, Chicago, USA, 1/200), total TDP-43 (polyclonal rabbit-anti-TDP43-Ab 12892-1-AP, Proteintech, 1/200), GFAP (Abcam, Cambridge, UK, 1/5000), NO-synthetase (Santa Cruz biotechnologies, Santa Cruz, USA, 1/400), ChAT (Chemicon International, 1/500) and HuCD (Invitrogen Life Technologies, 1/500). After rinsing, fluorescently labeled appropriate secondary antibodies were added for 2 h. Immunohistochemical staining was visualized under an epifluorescence microscope (BX 41 Olympus, Belgium) with specific filter cubes (EX/DM/EM in nm) for blue (325-375/400/435-485), green (460-495/505/510-550) and red fluorescent probes (570-590/595/600-660). Images were recorded using Cell^F software on an XM10 (Olympus) camera.

For counting of motor neurons, the lumbar spinal cord was cut in 20 μm thick slices, with each 6^th^ slice placed on a slide (10 in total along the whole lumbar region). These were stained by Cresylviolet (Sigma). After taking photos of the ventral horns at 40x (10/mouse) with a Zeiss Imager M1 microscope (Zeiss), manual counting and analyses of the size of motor neurons was done with use of AxioVision (Zeiss). Six NTG mice, 5 ES *TDP-43 (A315T)* mice with normal food and 5 ES *TDP-43 (A315T)* mice with gel food were analyzed.

Staining and quantification of the dorsolateral corticospinal tract axons in the distal thoracic spinal cord (of 4 NTG, 4 ES *TDP-43 (A315T)* mice on normal food and 4 ES mice on gel food) was done as described by Wegorzewska et al. [15].

### Western blot and qPCR

Spinal cord and brain from presymptomatic *TDP-43 (A315T)* or NTG mice (n = 3 for each genotype) were collected in Tripure isolation reagent (Roche Diagnostics, IN, USA) or tissue protein extraction reagents (Thermo Scientific, Rockford, IL, USA) with Complete (Complete EDTA-free, Roche Diagnostics) for qPCR and Western blot respectively.

RNA extraction was performed as described previously [36]. qPCR assays were run in triplicate with n = 3 for each condition. The mRNA expression in spinal cord and brain of mouse TDP-43 (Mm.PT.51.5553804 Tardp exon 2–3, IDT, Coralville, Iowa, USA) was compared to the expression of several housekeeping genes, using taqman assays: beta-actin (VIC-MGB 4352341E-0905010, Applied Biosystems, Life Technologies, CA, USA), mouse hypoxanthine-guanine phosphoribosyltransferase (Mm01545399_m1 Hprt, Applied Biosystems) and mouse glyceraldehyde 3-phosphate dehydrogenase (GAPDH) (Mm.PT.39a.1 GAPDH exon 2–3, IDT, Leuven, Belgium). Expression of mouse Sort1 + Ex17b mRNA in the brain of *TDP-43 (A315T)* and NTG mice was determined using Cyber green. The primers were: mouse Sort1 + Ex17b: 5′-AAATCCCAGGAGACAAATGC-3′ and 5′-GAGCTGGATTCTGGGACAAG-3′; mouse GAPDH: 5′- TGGCCTTCCGTGTTCCTAC -3′ and 5′- GAGTTGCTGTTGAAGTCGCA -3′.

For Western blot, 40 μg of spinal cord of *TDP-43 (A315T)* or NTG mice (n = 3 for each condition) were blotted using a precast gel (NuPAGE®Bis-Tris gel IM-8042, Novex, Life technologies, Carlsbad, California, USA) and following the protocol described in Van Hoecke et al. [37], allowing separation of the mouse TDP-43 (of 43 kDa) and the exogenous human TDP-43, with the Flag-tag running at a slightly higher molecular weight as described in Wegorzewska et al. [15]. The membranes were probed with polyclonal rabbit-anti-TDP43-Ab (12892-1-AP, Proteintech, 1/500). The mouse TDP-43 levels were compared between *TDP-43 (A315T)* mice and NTG mice. Alfa-tubulin (mouse anti-alfa-tubulin, T6199, Sigma, 1/5000) was used as a loading control. Also, the total TDP-43 expression between 90 days old *TDP-43 (A315T)* males and females was compared by using the polyclonal rabbit-anti-TDP43-Ab (12892-1-AP, Proteintech, 1/500) and normalized against mouse GAPDH (mouse anti-GAPDH AM4300, Ambion, Life Technologies, 1/5000).

### Statistics

Data are presented as mean ± standard error of the mean, except for Figure 3E and F in which the standard deviations are shown. All statistics were performed using Graph Path Prism or Stats Direct software. In case of not normally distributed values, a non parametric test was used to compare 2 groups. For more than 2 groups, an Anova or Kruskal-Wallis (in case of not normally distributed data) was used. Survival data were analyzed using the log-rank test.

## Abbreviations

TDP-43: TAR DNA-binding protein 43; ALS: Amyotrophic lateral sclerosis; FTLD: Frontotemporal lobar degeneration; NTG: Non-transgenic; CK: Creatine kinase; ES: Endstage; GFAP: Glial fibrillary acid protein; Prnp: Prion promoter; NOS: Nitric-oxide synthase; ChAT: Choline acethyltransferase; NMJ: Neuromuscular junctions; ENS: Enteric nervous system; Sort1 + ex17b: Sortilin 1 including exon 17b.

## Competing interests

The authors declare that they have no competing interests. All experiments on rodents were approved by the local ethical committee of the University of Leuven, Belgium.

## Authors’ contributions

SH carried out the breeding, genotyping, perfusions, qPCRs, Western blots, motor assessment, immunoassays and histopathology of the spinal cord and NMJs of the mice, as well as the quantifications and statistical analysis of these results. SH drafted the manuscript. CC has helped substantially to acquisition, analysis and interpretation of data on intestinal assays and in drafting the manuscript. PVD and PVB participated in the design and coordination of the studies and helped to analysis and interpretation of data and in drafting the manuscript. LVDB and WR have been involved in revising the manuscript. All authors read and approved the manuscript.

## Authors’ information

Pieter Vanden Berghe and Philip Van Damme share last senior authorship.

## Supplementary Material

Additional file 1 **Movies 1–4: Motor phenotype of symptomatic and ES *TDP-43 (A315T) *** **mice.** Difference in gait abnormalities between the *TDP-43 (A315T)* mouse on normal food (additional movie 1.vc), compared to one on gel food (additional movie 2.vc). The swimming gait of the gel fed mouse is much more pronounced and slowly proceeds till an ES situation, as is visible in movie 4 (additional movie 4.MOV). The mouse is not able to turn from its side anymore due to the severe neurologic impairment, whereas the *TDP-43 (A315T)* mouse on normal food in movie 3 (additional movie 3.MOV) is about to die due to intestinal pseudo-obstruction. Click here for file
